# An integrative study on the green cultural industry and its determinants in Jiangsu province, China under the cultural revitalization initiative: a global perspective

**DOI:** 10.3389/fpsyg.2024.1328121

**Published:** 2024-11-12

**Authors:** Yaoyao Ding, Rong Zhang, Yuntao Zou

**Affiliations:** ^1^School of Design and Art, Communication University of Zhejiang, Hangzhou, China; ^2^Nanjing University of the Arts, the Purple Academy of Culture and Creativity, Nanjing, China; ^3^Future Front Interdisciplinary Research Institute, Huazhong University of Science and Technology, Wuhan, China; ^4^School of Computer of Science and Technology, Huazhong University of Science and Technology, Wuhan, China; ^5^School of Energy and Power Engineering, Huazhong University of Science and Technology, Wuhan, China

**Keywords:** culture industry, sustainability, economic efficiency, influencing factors, global perspective

## Abstract

**Introductions:**

The cultural industry is pivotal in promoting sustainable economic development. This study aims to evaluate the economic efficiency of the cultural industry in Jiangsu Province by establishing a Data Envelopment Analysis (DEA) model and analyzing influencing factors using the Tobit model, all within the broader context of China’s cultural industry.

**Methods:**

A DEA model was developed to assess the economic efficiency of the cultural industry across Chinese provinces, allowing for a comparative analysis of performance. The Tobit regression model was utilized to investigate factors influencing these efficiency outcomes, with a particular emphasis on inter-provincial comparisons to understand the position and challenges faced by Jiangsu’s cultural industry.

**Results:**

Despite Jiangsu’s cultural industry ranking among the largest in scale nationally, its economic efficiency is only moderate, consistently experiencing diminishing returns to scale. The study identifies low scale efficiency, small enterprise size, and suboptimal urbanization processes within the province as the main issues.

**Discussion:**

The inefficiencies highlighted by the DEA model suggest a misalignment between the scale of operations and the economic outputs in Jiangsu’s cultural industry. Urbanization emerges as a crucial factor, with current practices not sufficiently supporting the growth potential of the cultural sector.

**Conclusion:**

Based on these findings, the study proposes targeted policy recommendations for Jiangsu, including avoiding blind scale expansion, adjusting industrial structures, encouraging enterprise consolidation and optimization, and identifying new growth areas to better support the cultural industry’s development and contribute to sustainable economic progress.

## 1 Introduction

Sustainable development traditionally hinges upon three fundamental dimensions: “Environmental, Social, and Economic.” In recent times, the inclusion of “Culture” as a fourth dimension has gained increasing advocacy, especially within the contexts of globalization and multiculturalism. Brocchi (2010) argue that the cultural dimension serves as a pivotal safeguard when addressing sustainability and is key to addressing global challenges. Duxbury et al. (2017) identify four roles that cultural policies can play in sustainable development: first, the protection and preservation of cultural traditions and rights; second, the operation and impact of green cultural organizations and industries; third, enhancing public awareness of sustainable development and climate change, promoting actionable steps; and fourth, fostering ecological citizenship.

The cultural industry, positioned at the nexus of cultural and economic dimensions, is typically perceived as an integral part of sustainable development, constituting a vital segment of the green economy. This is largely due to its reliance on creativity, knowledge, and skills rather than traditional natural resources or large-scale material production. It commonly exhibits the following characteristics:

Low carbon emissions: Compared to heavy industries and other material-intensive sectors, the carbon footprint of the cultural industry is relatively low. Activities such as design, coding, writing, music production, and filmmaking generally do not generate significant carbon emissions.

Limited resource independence: Unlike industries like mining or agriculture, the cultural industry does not directly rely on finite natural resources. While the manufacturing of CDs, books, or film equipment might require material resources, the core value lies in the creative content, not the physical medium.

Promotion of sustainability ideals: The cultural industry, especially the media and entertainment sectors, wields substantial influence and has the capacity to convey messages about environmental conservation and sustainable development to the public.

Driving innovation: The cultural industry encourages innovative thinking and creative approaches, which can be applied to broader areas, including green technologies and sustainable development solutions.

Promoting a circular economy: Cultural products such as digital content, music, films, and books can be consumed repeatedly without leading to resource depletion or wastage.

Providing green employment: The cultural industry offers employment opportunities for many, and these jobs are often greener and more sustainable compared to traditional “blue-collar” positions.

In conclusion, the development of the cultural industry has profound significance for the economic, environmental, and social dimensions of sustainable development. By promoting the growth of the cultural industry, not only can economic expansion be spurred, but also environmental protection can be enhanced, societal cohesion and cultural identity can be strengthened, and cultural diversity and innovation can be fostered, all contributing to global sustainable development.

## 2 Literature review and objectives of this study

### 2.1 Studies pertaining to the cultural industry

The nexus between culture and sustainable development is intricate and multifaceted, manifesting in the interconnectedness among social, economic, and environmental sustainability. The cultural industry aids in preserving and promoting diverse cultures and traditions, ensuring cultural diversity and continuity. Research by Nurse (2006) suggests that culture serves as the fourth pillar of sustainable development, with the cultural industry being a focal point. In their exploration of the Baltic economy, Klein et al. (2021) discerned that cultural creativity is both a driver and an impetus for human sustainable progression. It empowers individuals to take responsibility for their own evolution, spurring innovation and creativity, thereby propelling inclusive and sustainable advancement.

The cultural industry is a pivotal driver of economic growth, fostering creativity and innovation which, in turn, can propel other sectors such as technology and manufacturing toward sustainable economic development. A study by Vila et al. (2021) elucidates that sustainable development is intrinsically linked to the cultural policies of the European Union. Of the 17 Sustainable Development Goals (SDGs) defined by the United Nations, eight encompass cross-disciplinary themes related to culture: cultural and natural heritage (SDG 11), cultural education (SDG 4), cultural diversity (SDG 4), social inclusion (SDG 10), access to information (SDG 16), partnerships with other institutions (SDG 17), environment (SDG 13), economic growth (SDG 8), and gender equality (SDG 5). d'Orville (2019) posit that creativity, at the heart of sustainability, represents a distinct renewable resource and is emblematic of human prowess. The cultural industry stands as one of the central proponents enhancing this creativity, with the creative economy epitomizing the most modern phase of economic evolution.

In conclusion, the value of the cultural industry is increasingly recognized. It demonstrates sustainability not only in economic terms but also closely intertwines with the SDGs on social and environmental fronts. To fully harness its potential, collaborative efforts across various sectors are imperative, ensuring the cultural industry genuinely becomes a vital force propelling sustainable development.

### 2.2 Characteristics of the cultural industry in China

China places significant emphasis on the development of its cultural industry. On July 22, 2009, China’s first specialized plan for the cultural sector, titled “Cultural Industry Revitalization Plan,” was approved by the State Council’s executive meeting (China, 2009). This signified the elevation of the cultural industry to a strategic national level. In 2017, China introduced the “13th Five-Year Plan” for cultural development (China, 2017), explicitly stating the objective to establish a modern cultural industry system. The newly issued “14th Five-Year Plan” for cultural development in 2022 positions cultural construction prominently in overall national work, emphasizing it as a crucial task for enhancing national cultural confidence, promoting the modernization of excellent traditional Chinese culture, showcasing the allure of Chinese culture, and opposing cultural hegemony. In 2018, the former Ministry of Culture and the National Tourism Administration were officially merged to form the Ministry of Culture and Tourism, thereby formally integrating tourism into the cultural industry.

Research conducted by Shan et al. indicates that as Western nations increasingly recognize cultural development as a new source of economic growth, China has also placed a growing emphasis on the development of its cultural Industry, with the scale and magnitude expanding annually (Shan, 2014). The contributions of the cultural Industry to the socio-economic landscape have been on the rise, with production capabilities witnessing significant enhancement. The integration of culture with technology has become more intricate than before, with the emergence of novel industries filling gaps in the market. The establishment of financing systems and the development of cultural resources have become more systematic. Industry clusters have started trending, and there has been an uptick in cultural exports, leading to a thriving cultural trade environment and an unprecedentedly vibrant cultural market.

Vlassis, in a study on Chinese film production and its market, ascertained that China’s cultural Industry have started to exhibit global competitiveness (Vlassis, 2016). Ju et al., employing the DEA (Data Envelopment Analysis) methodology to assess the efficiency of China’s cultural manufacturing sector, advocated for the encouragement, support, and guidance of social capital to intensify investments in the cultural domain (Ju, 2019).

### 2.3 Studies related to the cultural industry of Jiangsu province

Jiangsu is a major economic province in China. It boasts a rich legacy of historical and cultural heritage. The gross output value of Jiangsu’s cultural industry exceeds 5% of the province’s total GDP (China, 2015). Research by He (2017) indicates that the local distinctive cultural industry of Jiangsu is set to become an integral component of the region’s economic growth. Lin et al. (2022) contend that as a powerhouse in cultural economy, Jiangsu’s future focus should lie in the optimal integration of traditional and emerging cultural Industry. It’s imperative to enhance the innovative awareness across the cultural sector, proactively develop high-end, customized cultural products, create products to generate demand, and innovate to attract demand. Following an analysis of cultural industry clusters in 13 cities within Jiangsu, Feng et al. (2023) observed that the province’s cultural industry development surpasses the national average, though there exists a significant disparity in the development levels across the 13 cities.

### 2.4 Research objectives and hypotheses

Empirical research on regional economics often begins with data from the sub-regions or constituent parts of the main region to evaluate its overall economic status. For instance, when Li et al. assessed the firefighting standards in the United States (Li et al., 2018), they based their evaluation on data from individual states. Similarly, when Su et al. (2019) analyzed the sustainability of China’s culture and tourism industry by evaluating the efficiency of each province. In their exploration of the correlation between fiscal support and agricultural circular economic efficiency in Henan Province, Chen et al. (2023) utilized data from various prefecture-level cities within Henan to gauge the overall level of its agricultural economy. Feng et al. (2023) also assessed the development level of Jiangsu’s cultural industry by examining the cultural sectors across 13 prefecture-level cities in Jiangsu.

However, the subjects of research are often not isolated and need to be placed in a broader context for comparative studies with similar entities to identify disparities and analyze reasons, thereby gaining a more comprehensive understanding. The goal of this study is to investigate the cultural industry of Jiangsu Province from a national perspective, by analyzing panel data and influencing factors from all 31 provinces and cities in China, to assess the position of Jiangsu’s cultural industry nationally, identify existing issues, and propose targeted recommendations. Consequently, this study posits the following hypotheses:

Hypothesis 1: Jiangsu Province, with its rich cultural heritage and strong economic foundation, holds a leading position in the scale of its cultural industry.

Hypothesis 2: The development level of Jiangsu’s cultural industry surpasses that of other provinces in China.

Hypothesis 3: The current comprehensive policies in Jiangsu Province are favorable for the development of the cultural industry.

## 3 Materials and methods

### 3.1 Materials

This study is grounded upon the panel data of cultural Industry from 31 provinces and municipalities in China spanning from 2018 to 2021. While China consists of 34 provincial administrative units, the present research incorporates data from 31 provinces, municipalities, and autonomous regions. Three regions, namely Taiwan, Hong Kong, and Macau, are excluded due to considerable disparities in data collection, policy implementation, and historical contexts when juxtaposed with the selected 31 areas, rendering them unsuitable for the study’s scope.

Data sources: China Statistical Yearbook, China Cultural Industry Statistical Yearbook, and China Tax Statistical Yearbook.

### 3.2 Methods

This study evaluates the cultural industry using Data Envelopment Analysis (DEA), conducting efficiency assessments of the cultural industry across various provinces nationwide. DEA was introduced by Charnes et al. (1978). In their study, they employed linear programming techniques to address the issue of evaluating the efficiency of a specific entity. Within the DEA framework, multiple entities under evaluation are termed as Decision Making Units (DMUs), with each DMU having consistent input and output variables. An Envelope Surface, constituting a convex polygon, is then constructed to assess the efficiency of DMUs, with this polygon being capable of entirely encompassing all DMUs. DMUs located on the Envelope Surface are considered DEA-efficient, receiving an efficiency score of 1, while the relative efficiency scores for the remaining DMUs are determined based on their distance to the Envelope Surface. Due to its non-parametric nature and the absence of a requisite to preset weights, DEA has garnered increasing attention and its application has expanded across various domains and industries.

The foundational DEA models are the CCR and BCC models. Both the CCR and BCC models consider only radial improvements and neglect the slackness of variables. Their envelope surfaces are convex polygons, hence not fully optimized. In reality, an optimal envelope surface might exhibit non-convexity or non-smoothness. Addressing this, Tone et al. introduced the SBM model in 2001, integrating ‘slack variables’ to measure the ‘slackness’ of each DMU in input–output variables, creating a more optimized envelope surface (Tone, 2001). The SBM model offers advantages in practical applications, allows decomposition of efficiency scores into pure technical efficiency and scale efficiency, and provides slacks for each variable, expanding its interpretability.

Ohsato and Takahashi utilized the DEA-SBM model and further expanded it to the Network SBM model when assessing the management efficiency of banks in Japan (Ohsato and Takahashi, 2015). Cecchini et al. applied the SBM model with undesired outputs to evaluate the green production efficiency of dairy farms in Italy (Cecchini et al., 2018). In the realm of cultural Industry, Su et al. employed the SBM model to evaluate China’s culture and tourism sectors (Su et al., 2019). Similarly, Gao and colleagues utilized the SBM model in their research on the tourism efficiency of Chinese provinces (Gao et al., 2022).

The methodology of this study is based on the SBM model. The equation for SBM can be presented as follows (Tone, 2011):

In the input-oriented Slacks-Based Measure (SBM) model, the objective is to minimize slack variables or equivalently maximize efficiency, subject to constraints on inputs and outputs. To assess the relative efficiency of $DMU_{o}=\left(x_{o},y_{o}\right)$ , the following linear programming formulation can be solved. This process is repeated n times for $o=\left(1,\ldots ,n\right)$ .

[SBM-I-C] (Input-oriented SBM under constant returns-to-scale assumption) Formally, the input-oriented SBM model can be articulated as the following linear programming problem:

Objective Function:


$$\rho_{I}^{*}=\min_{\lambda ,s^{-},s^{+}}\;1-\frac{1}{m}\overset{m}{\underset{i=1}{\sum }}\frac{s_{i}^{-}}{x_{\mathrm{io}}}$$

Subject to:


$$x_{\mathrm{io}}=\overset{n}{\underset{j=1}{\sum }}x_{ij}\lambda_{j}+s_{i}^{-}\;\left(i=1,\ldots ,m\right)$$

$$y_{ro}=\overset{n}{\underset{j=1}{\sum }}y_{rj}\lambda_{j}-s_{r}^{+}\;\left(r=1,\ldots ,s\right)$$

$$\lambda_{j}\geq 0\left(\forall j\right),\;s_{i}^{-}\geq 0\left(\forall i\right),\;s_{r}^{+}\geq 0\left(\forall r\right)$$

$\rho_{I}^{*}$ is called SBM-input-efficiency.

However, the CCR, BCC, and SBM models are all established based on cross-sectional data from the same period to derive relative efficiency scores. When dealing with panel data from different periods, these models, owing to their varying reference sets and envelopment surfaces across periods, do not allow for direct comparison of the efficiency scores of the same Decision Making Unit (DMU) across different times. Therefore, Golany and Roll (1989) introduced the Global DEA model, treating DMUs across all periods as a unified reference set for evaluation. This approach enables the study of how efficiency scores change over time and facilitates direct comparisons between different periods. The Global DEA model offers convenience in analyzing efficiency trends and evolution. Moreover, it provides a more rational foundation for regression analysis, circumventing some of the issues associated with the Malmquist index. Streimikis et al. (2021) employed the Global DEA model to assess the energy efficiency of agriculture in the European Union. Ben Lahouel et al. (2023), in their study of inclusive green growth in OECD member countries, utilized the Global Malmquist index. Zhao et al. (2022) incorporated the Global Meta-frontier SBM Super-efficiency model in their research on the impact of environmental regulations on green economic growth in China.

This study employs regression analysis for its correlational research. Regression analysis, a statistical method for quantifying the interdependent relationships among two or more variables, was introduced in the 19th century. With advancements in mathematics and statistics, nearly a hundred commonly used models have been developed. Various regression models provide predictive, inferential, causal, and trend analyses, aiding decision-makers in optimizing strategies, thus widely applied in fields such as economics, biology, engineering, social sciences, and business. Cao et al. (2024) used a two-way fixed effects regression model in their study of the impact of geopolitics on natural resources. Wu et al. (2024) utilized the Panel Smooth Transition Regression model to investigate the effects of monetary policy on carbon emissions. Guo et al. (2023) applied a combined DEA-Tobit regression model to explore the impact of China’s rural revitalization strategy on the agricultural economy.

Utilizing the efficiency scores from the Global SBM model as the dependent variable, a regression analysis was performed in conjunction with selected influential factors. The efficiency scores derived from the Global DEA-SBM model range between 0 and 1. In traditional linear regression, the dependent variable can assume any real value. When dealing with such truncated data, this may lead to significant model errors. James Tobin introduced the Tobit regression model in Tobin (1958), aiming to more accurately estimate regression model parameters by accounting for such truncation or limitation. The Tobit regression model serves as a statistical regression approach tailored for truncated or censored dependent variables. Given that this study employs efficiency scores from the Global SBM model as the dependent variable—a value constrained between 0 and 1—it is aptly suited for analysis using the Tobit regression model.

### 3.3 Research process

**Figure 1:**
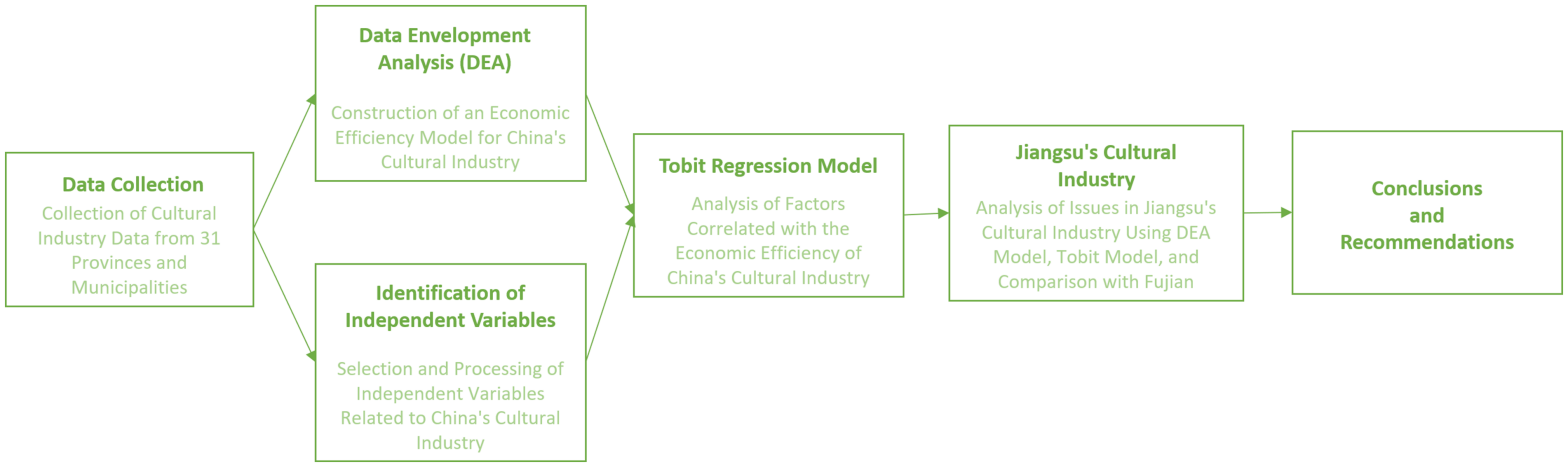
Research methodology flowchart of this study.

The research process adopted in this study is illustrated in Figure 1: Initially, panel data from the cultural Industry of 31 provinces and municipalities in China from 2018 to 2021 is employed to construct the global SBM model. Subsequently, using the acquired technical efficiency as the dependent variable and integrating selected independent variables, a Tobit regression model is established. Based on the outcomes from both models, an analysis is conducted on the economic efficiency of the cultural industry nationally, with a specific focus on Jiangsu Province. Conclusions are drawn, and targeted recommendations are subsequently proposed.

## 4 Results

### 4.1 Global SBM model for economic efficiency of China’s cultural industry, 2018–2021

For the sake of data integrity, this study selected “Number of Enterprises at Year-end,” “Total assets at Year-end,” and “Engaged Persons at Year-end” as input variables, with “Total Revenue” serving as the output variable. The decision to not incorporate additional variables stemmed from concerns over potential multicollinearity among similar variables. Excessive variables might also introduce increased statistical noise, both of which could compromise the precision of the model. The input and output variables adopted in the model are presented in Table 1.

**Table 1:** List of inputs and outputs.

Indicator categories	Indicators
Input indicators	Number of enterprises at year-end
	Total assets at year-end
	Engaged persons at year-end
Output indicators	Total revenue

This study employed DEARUN software version 3.2.0.2 Trial to construct a global SBM model assessing the economic efficiency of the cultural industry across China’s 31 provinces from 2018 to 2021. The average efficiency scores for these 4 years across the provinces are summarized in Table 2, with complete results detailed in the Supplementary Table S1. In this model, TE represents Technical Efficiency under the assumption of constant returns to scale, PTE denotes Pure Technical Efficiency under variable returns to scale, and SE stands for Scale Efficiency, where a value of 1 indicates DEA effectiveness. In the ‘Return to Scale’ column, ‘CRS’ indicates constant returns to scale for the respective province or city, ‘IRS’ indicates increasing returns, and ‘DRS’ denotes decreasing returns. It is important to note that within the DEA-SBM model, TE is the product of PTE and SE.

**Table 2:** Summary of results from the 2018–2021 economic efficiency model for China’s cultural industry.

	Mean value of TE	Mean value of PTE	Mean value of SE	Number of CRS	Number of DRS	Number of IRS
2018	0.36318725	0.445118065	0.886811874	6	20	5
2019	0.39392756	0.480749075	0.896238444	7	19	5
2020	0.382403556	0.45110521	0.896912632	7	19	5
2021	0.440109884	0.551576374	0.866256087	8	18	5

### 4.2 The Tobit regression model for economic efficiency of the cultural industry in China

#### 4.2.1 Determination of independent variables for the Tobit regression model

Through data collection, this study identified a total of 7 independent variables. Specifically, they are:

##### 4.2.1.1 Percentage of *per capita* expenditure on culture and recreation

This variable is calculated as the annual *per capita* expenditure on culture and recreation relative to the overall *per capita* expenditure, and it is expressed in percentages (%). This represents the level of cultural and entertainment consumption in each province or city. Data for this variable was sourced from the China Statistical Yearbook and the China Cultural Statistics Yearbook.

##### 4.2.1.2 *Per capita* disposable income

Measured in units of 10,000 CNY, this variable signifies the affluence level of each province or city. The data was extracted from the China Statistical Yearbook.

##### 4.2.1.3 *Per capita* gross regional product

Expressed in units of 10,000 CNY, it represents the economic level of each province or city. Data for this metric was directly sourced from the China Statistical Yearbook.

##### 4.2.1.4 Proportion of urban population

Expressed in percentages (%), this metric reflects the urbanization level of each province or city. Data for this variable is sourced from the China Statistical Yearbook.

##### 4.2.1.5 Percentage of assets of large-scale cultural industry enterprises

Presented in percentages (%), it characterizes the extent of scaling of cultural industry-related enterprises in each province or city. It is calculated by dividing the total assets of large-scale cultural industry enterprises in each province or city by the total assets of cultural industry enterprises in that province or city. This data is drawn from the China Cultural Statistics Yearbook.

##### 4.2.1.6 Tax-to-asset ratio: indicated in percentages

Expressed in percentages (%), this variable signifies the tax burden of the cultural industry in each province or city. It’s calculated by taking the sum of total cultural industry-related taxes in each province or city and dividing it by the total assets of cultural industry enterprises in that province or city. The data is derived from the China Statistical Yearbook and the China Taxation Statistical Yearbook.

##### 4.2.1.7 Degree of financial support

Represented in percentages (%), this variable indicates the level of support each province or city provides to its cultural industry. It is computed by dividing the expenditure by the provincial or city government on Culture, Sport, and Media by the general public budget expenditure of that province or city. The data is sourced directly from the respective yearbooks. It’s important to note that the expenditure for sports is included in this data. Since there’s no specific breakdown available for sports-related expenses, using the combined figure for “Expenditure for Culture, Sport, and Media” might impact model precision to some extent. However, since all 31 provinces or cities include sports expenditures in their data, and the proportion tends to be relatively consistent, the actual impact on model accuracy is minimal. Therefore, this variable has been retained in the study.

#### 4.2.2 Constructing the Tobit model for factors influencing the economic efficiency of China’s cultural industry from 2018 to 2021

In this study, SPSSAU software was employed to establish a Tobit regression model. The technical efficiency score TE from the global SBM model of the cultural industry in China’s 31 provinces from 2018–2021 was taken as the dependent variable with a left truncation boundary of 0 and a right truncation boundary of 1.1. Seven independent variables were included: *Per capita* expenditure on culture and recreation, *Per Capita* Disposable Income, Proportion of assets of cultural industry enterprises above scale, *Per Capita* Gross Regional Product, Proportion of Urban Population, Expenditure for Culture, Sport and Media, and Total Tax.

The likelihood ratio test results of the Tobit model are presented in Table 3. The *p*-value of the model is less than 0.05, which implies the rejection of the null hypothesis, signifying that at least one independent variable has a significant influence on the dependent variable, thus validating the model’s effectiveness. The Akaike Information Criterion (AIC), named after the Japanese statistician Hirotsugu Akaike (Akaike, 1974), serves as a measure to compare the fit and complexity of different models, with a lower value being preferable. The Bayesian Information Criterion (BIC), proposed by Schwarz (Schwarz, 1978), is employed to guard against overfitting due to excessive model complexity. The likelihood ratio test for this model yields relatively low AIC and BIC values, indicating a commendable relative validity of the model.

**Table 3:** Results of Tobit model likelihood ratio test.

Model	−2 × log-likelihood	Cardinality	*df*	*p*	AIC	BIC
Intercept distance	−18.686				−18.686	
Final model	−138.275	119.588	7	0.000	−122.275	−99.712

The final results of the Tobit model are summarized in Table 4.

**Table 4:** Results of Tobit model.

	Regression coefficient
*Per capita* expenditure on culture and recreation as a percentage (%)	−0.017 (−1.053)
*Per capita* disposable income (10,000 CNY)	0.086* (2.534)
Per of assets of culture industry enterprises above scale (%)	0.004** (3.624)
*Per capita* gross regional product (10,000 CNY)	0.050** (4.353)
Proportion of urban population (%)	−0.017** (−7.310)
Degree of financial support (%)	−0.051 (−1.666)
Tax-to-asset ratio (%)	0.156 (1.578)
likelihood ratio test	χ^2^(7) = 119.588, *p* = 0.000
McFadden *R*^2^	−6.400
Implicit variable: TE	

*p < 0.05, **p < 0.01, *z*-values in parentheses.

## 5 Discussions

### 5.1 Economic efficiency development and its influencing factors in China’s cultural industry from 2018 to 2021

**Figure 2:**
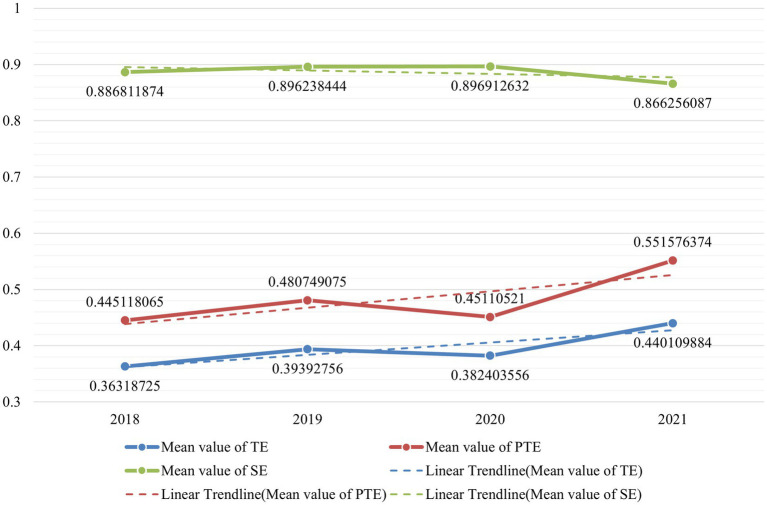
Mean efficiency and development trend of China’s cultural Industry global SBM model from 2018 to 2021.

In Section 4.1 of this study, the constructed Global SBM model is suitable for cross-period comparisons. By summarizing the mean values of TE, PTE, and Scale for the 31 provinces and cities from Table 2, we present the results in Figure 2. As illustrated in Figure 2, the efficiency of the cultural industry in China’s 31 provinces and cities shows an upward trend from 2018 to 2021. Although there was a slight decline between 2019 and 2020 - which is clearly attributable to the consequences of the Covid-19 pandemic - the efficiency of the cultural industry rebounded in the trend from 2020 to 2021, recovering the previous year’s decline.

Figure 2 also reveals that the Scale Efficiency is relatively high across the 31 provinces and cities, while Pure Technical Efficiency is lower. In the DEA-SBM model, TE is the product of PTE and SE. This suggests that the production scale of China’s cultural industry has reached a relatively good level, but the main constraint on economic efficiency is the pure technical efficiency, i.e., the PTE score, which includes factors beyond industrial scale such as management and technological levels. However, from the linear trend lines, it is evident that improvements in PTE scores are driving increases in TE scores, particularly between 2020 and 2021. This indicates significant advancements in aspects beyond scale, such as technological, management, and investment levels in China’s cultural industry.

**Figure 3:**
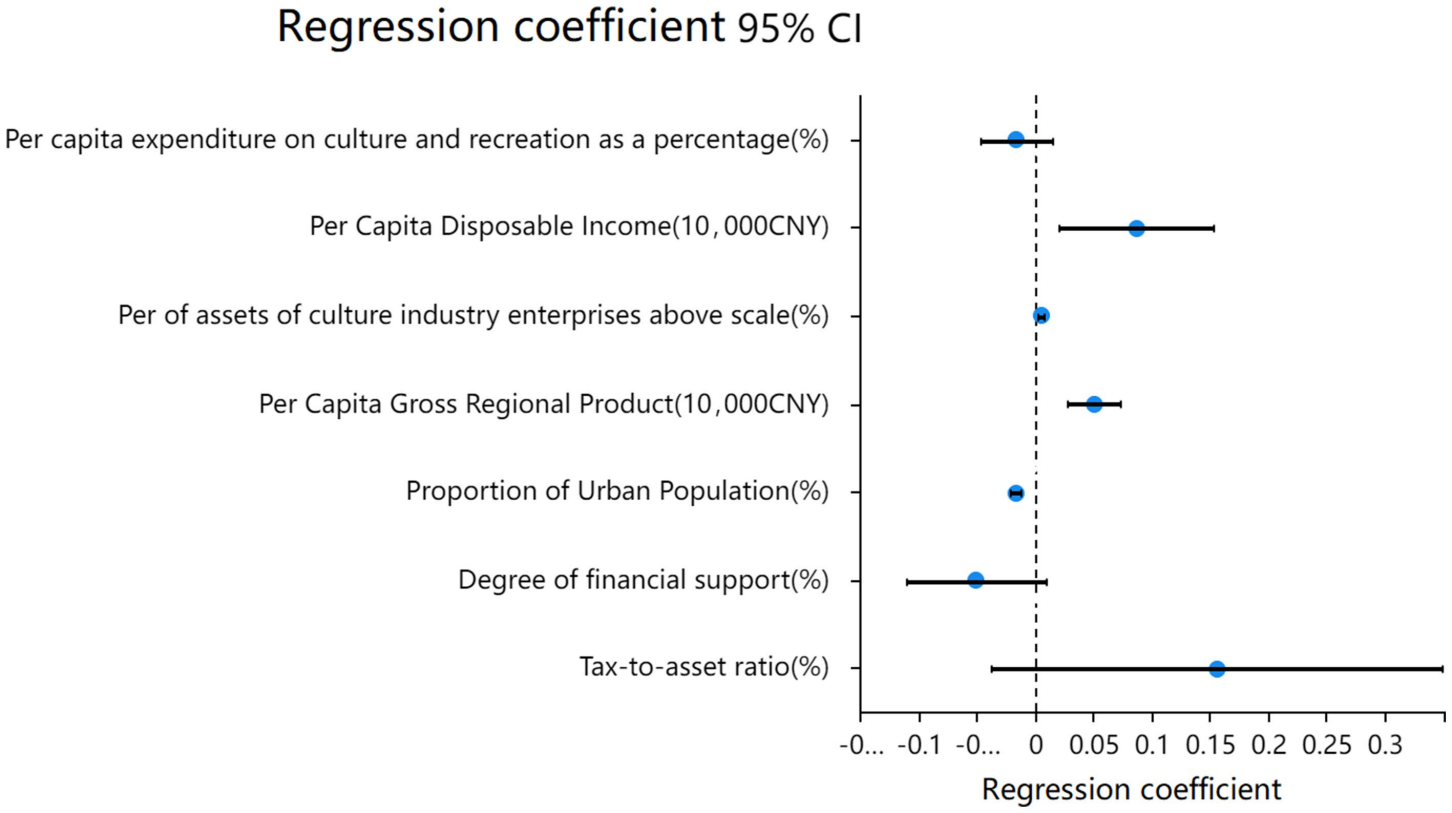
2018–2021 Tobit regression model of economic efficiency of China’s cultural industry.

The Tobit model from Table 4 can be represented as a forest plot, as depicted in Figure 3.

The equation for the Tobit model of China’s cultural industry economic efficiency can be expressed as:


$$\begin{array}{c} TE=0.761-0.017\\ \times \mathrm{Per}\;\mathrm{capita}\;\mathrm{expenditure}\;\mathrm{on}\;\mathrm{culture}\;\mathrm{and}\;\mathrm{recreation}\;as\;a\;\mathrm{percentage}\\ +0.086\times \mathrm{Per}\;\mathrm{Capita}\;\mathrm{Disposable}\;\mathrm{Income}+0.004\\ \times \mathrm{Per}\;\mathrm{of}\;\mathrm{assets}\;\mathrm{of}\;\mathrm{culture}\;\mathrm{industry}\;\mathrm{enterprises}\;\mathrm{above}\;\mathrm{scale}\\ +0.050\times \mathrm{Per}\;\mathrm{Capita}\;\mathrm{Gross}\;\mathrm{Regional}\;\mathrm{Product}-0.017\\ \times \mathrm{Proportion}\;\mathrm{of}\;\mathrm{Urban}\;\mathrm{Population}-0.051\\ \times \mathrm{Degree}\;\mathrm{of}\;\mathrm{financial}\;\mathrm{support}+0.156\times \mathrm{Tax}-to-\mathrm{asset}\;\mathrm{ratio} \end{array}$$

The regression coefficient for “*Per capita* expenditure on culture and recreation as a percentage” is-0.017, yet it is not statistically significant (*z* = −1.053, *p* = 0.292 > 0.05), suggesting that this variable does not exert a significant effect on TE.

On the other hand, the regression coefficient for “*Per Capita* Disposable Income” is 0.086, and it exhibits significance at the 0.05 level (*z* = 2.534, *p* = 0.011 < 0.05), indicating a positive significant relationship between this variable and TE. Similarly, the coefficient for “*Per Capita* Gross Regional Product” stands at 0.050, presenting significance at the 0.01 level (*z* = 4.353, *p* = 0.000 < 0.01). This indicates that “*Per Capita* Gross Regional Product” has a significant positive effect on TE. These independent variables reflect the economic level of the provinces and the affluence of the residents, hinting at increased consumption demands, superior management standards, a more favorable financing environment, and higher workforce quality. These factors, in turn, positively influence the economic efficiency of the cultural industry. Studies by Zeng et al. (2016) have similarly demonstrated this relationship, emphasizing that regional and residential economic levels significantly boost the efficiency of China’s cultural industry. Xie et al. (2022) also confirm these findings in their research.

The regression coefficient for “Per of assets of culture industry enterprises above scale” is 0.004, achieving significance at the 0.01 level (*z* = 3.624, *p* = 0.000 < 0.01). This indicates a significant positive relationship between this variable and TE. According to various publications by the Chinese government, enterprises with an annual primary business revenue exceeding RMB 20 million are classified as above-scale enterprises. This independent variable depicts the proportion of assets held by above-scale enterprises in the total assets of the cultural industry, representing the scale of cultural enterprises across provinces. Ju et al. while investigating Chinese port-listed companies, discovered a direct correlation between company size and efficiency (Ju and Liu, 2015). Research by Tho et al. revealed a noticeable increase in the economic benefits of Vietnamese agricultural breeding enterprises once they surpass a certain scale (Tho et al., 2021). Hassen et al. in their study on green economic efficiency, observed that larger companies are more inclined to adopt energy-saving and emission-reduction measures (Hassen et al., 2018). These studies underscore the positive influence of scale on production efficiency across various business sectors, and the cultural industry is no exception, especially with cultural manufacturing enterprises holding a significant proportion.

The regression coefficient for “Degree of financial support” is –0.051. However, it does not achieve statistical significance (z = −1.666, *p* = 0.096 > 0.05), suggesting that the “Degree of financial support” does not influence TE. Similarly, the regression coefficient for the “Tax-to-asset ratio” is 0.156, but it too fails to reach statistical significance (*z* = 1.578, *p* = 0.115 > 0.05), indicating that the “Tax-to-asset ratio” does not have a significant relationship with TE.

The regression coefficient for the “Proportion of Urban Population” stands at –0.017 and is statistically significant at the 0.01 level (*z* = −7.310, *p* = 0.000 < 0.01), indicating a significant negative influence on TE. This independent variable primarily reflects the urbanization level of each province. The results from this regression seem to contradict the previous findings about the impact of enterprise size on the efficiency of the cultural industry, since urbanization often leads to industrial agglomeration which benefits from economies of scale. However, larger cities often entail increased public service expenditures and higher tax burdens, leading to diminishing returns to scale. Mera et al., as early as 1970, found that if the objective of developing countries is to maximize the growth rate of GDP, urbanization clustering should be intensified (Mera, 1970). If a more equitable income distribution across regions is the goal, focus should be given to underdeveloped areas with decentralized powers, though this does not necessarily augment overall economic efficiency.

With the rise of sustainable development concepts in the 21st century, there has been a divergence in the impact of urbanization on economic efficiency, as cities tend to be more polluting than rural areas. Studies by Chang and Chen (2021) revealed potential risks posed by China’s rapid urbanization; economically advanced eastern regions have yet to achieve harmonious development between land economic benefits and the environment. Wang and Salman (2022) discovered that market-oriented environmental regulation stimulates green economic efficiency when the urbanization rate is below the first threshold and above the second. Between these thresholds, such regulation hinders green economic efficiency. These studies illustrate the potential negative impacts of urbanization on economic efficiency.

In conclusion, “*Per Capita* Disposable Income,” “Percentage of assets of culture industry enterprises above scale,” and “*Per Capita* Gross Regional Product” have a significant positive relationship with TE. Conversely, “Proportion of Urban Population” exhibits a significant negative relationship. “*Per capita* expenditure on culture and recreation as a percentage,” “Degree of financial support,” and “Tax-to-asset ratio” do not demonstrate a significant relationship with TE.

### 5.2 The economic efficiency of the cultural industry in Jiangsu is relatively low

**Figure 4:**
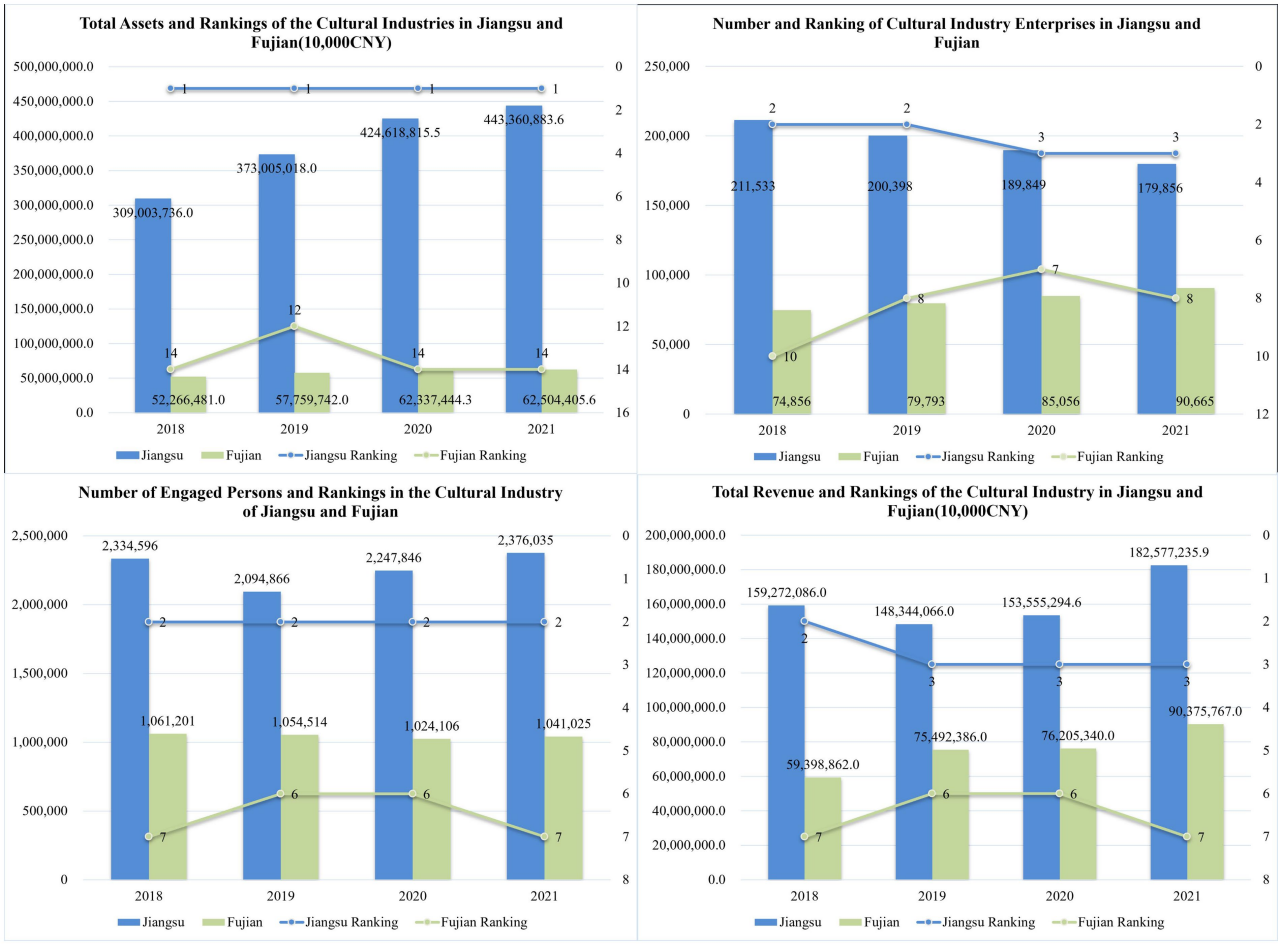
Input–output variable values and rankings for Jiangsu and Fujian from 2018 to 2021.

This study focuses on the cultural industry of Jiangsu and compares it with that of Fujian, which is also located in the economically developed southeastern coastal region of China. The values of three input variables and one output variable from the global SBM model for Jiangsu and Fujian, as well as their rankings among the 31 provinces and cities, are organized into Figure 4. As shown in Figure 4, in terms of input variables, Jiangsu’s cultural industry’s total assets have consistently ranked first nationwide for four consecutive years, with values continuing to rise. In contrast, Fujian’s cultural industry total assets are ranked between 12th and 14th, with a significantly weaker increase compared to Jiangsu. The ranking of the number of cultural industry enterprises in Jiangsu has consistently been high, ranging from 2nd to 3rd place, while Fujian ranks between 7th and 10th, with a slight increase in values. In terms of the number of employees in the cultural industry, Jiangsu maintains the second position nationwide, whereas Fujian ranks between 6th and 7th.

Regarding the output variables, as shown in Figure 4, the total revenue of Jiangsu’s cultural industry has consistently ranked between the 2nd and 3rd positions, while Fujian ranks between 6th and 7th, with a slight increase in values. In terms of the number of employees in the cultural industry, Jiangsu has steadily held the second position nationwide, whereas Fujian ranks between 6th and 7th. Taken together, these results support Hypothesis 1, which posits that Jiangsu’s cultural industry is among the foremost in the country. This prominence is likely due to Jiangsu being one of the most economically developed provinces in China with a rich traditional cultural heritage.

**Figure 5:**
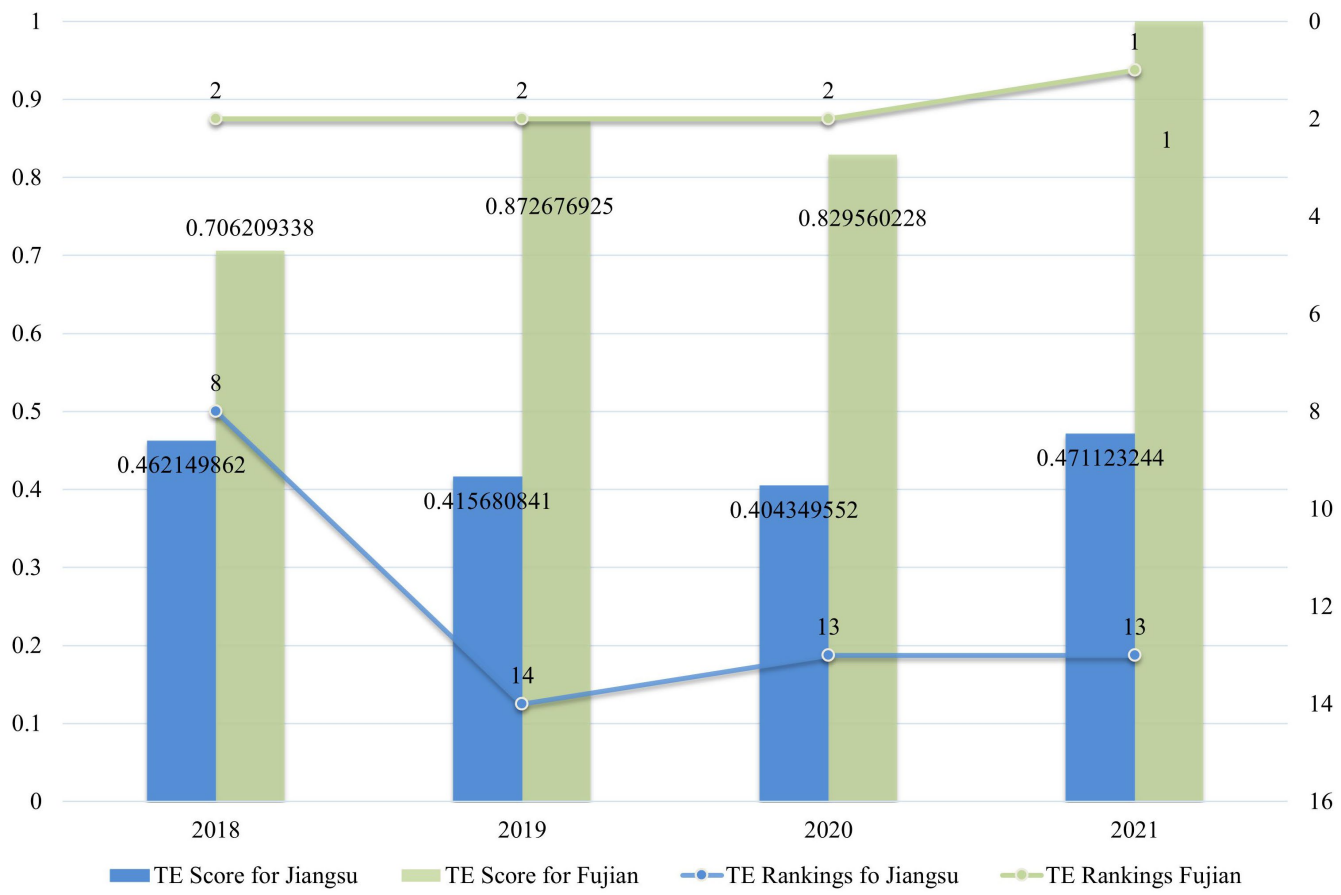
2018–2021 TE scores and rankings of the cultural industry in Jiangsu and Fujian.

The TE scores and rankings of the cultural industry in Jiangsu and Fujian are organized into Figure 5. This study uses a global SBM model, allowing for direct comparisons. As indicated in Figure 5, during the period from 2018 to 2021, the TE scores of Jiangsu’s cultural industry were all below 0.5, with significant declines in 2019 and 2020 compared to 2018. In the rankings among the 31 provinces and cities, Jiangsu was only ranked 8th in 2018, falling out of the top 10 in subsequent years. In contrast, Fujian’s TE scores have consistently ranked second nationwide, reaching DEA effectiveness in 2021, thus becoming one of the provinces with the highest scores in the country.

Based on the input–output variables selected for this study, Jiangsu’s cultural industry ranks among the top in the 31 provinces and cities nationwide, while Fujian maintains a mid-level position. However, the 31 provinces vary significantly in terms of area, geographical location, population, climate, and overall economic scale, so the industry scale alone does not represent the level of the cultural industry in each province. This study evaluates the cultural industry of various provinces through economic efficiency, where higher efficiency implies achieving more output with fewer inputs, allowing for a fairer assessment that mitigates regional disparities.

In conclusion, results from the global SBM model indicate that the technical efficiency of Jiangsu’s cultural industry ranks at a moderate level nationally, which does not correspond to its high input scale ranking, and there is no evident trend of ranking improvement. Therefore, Hypothesis 2 rejects the original assumption that Jiangsu’s cultural industry leads nationally; it only ranks in the mid-tier. For investors, the same resources might yield better returns if invested in other provinces. In contrast, Fujian, despite its average industry scale, ranks among the highest in national economic efficiency.

### 5.3 The scale returns to Jiangsu’s cultural industry are diminishing

**Figure 6:**
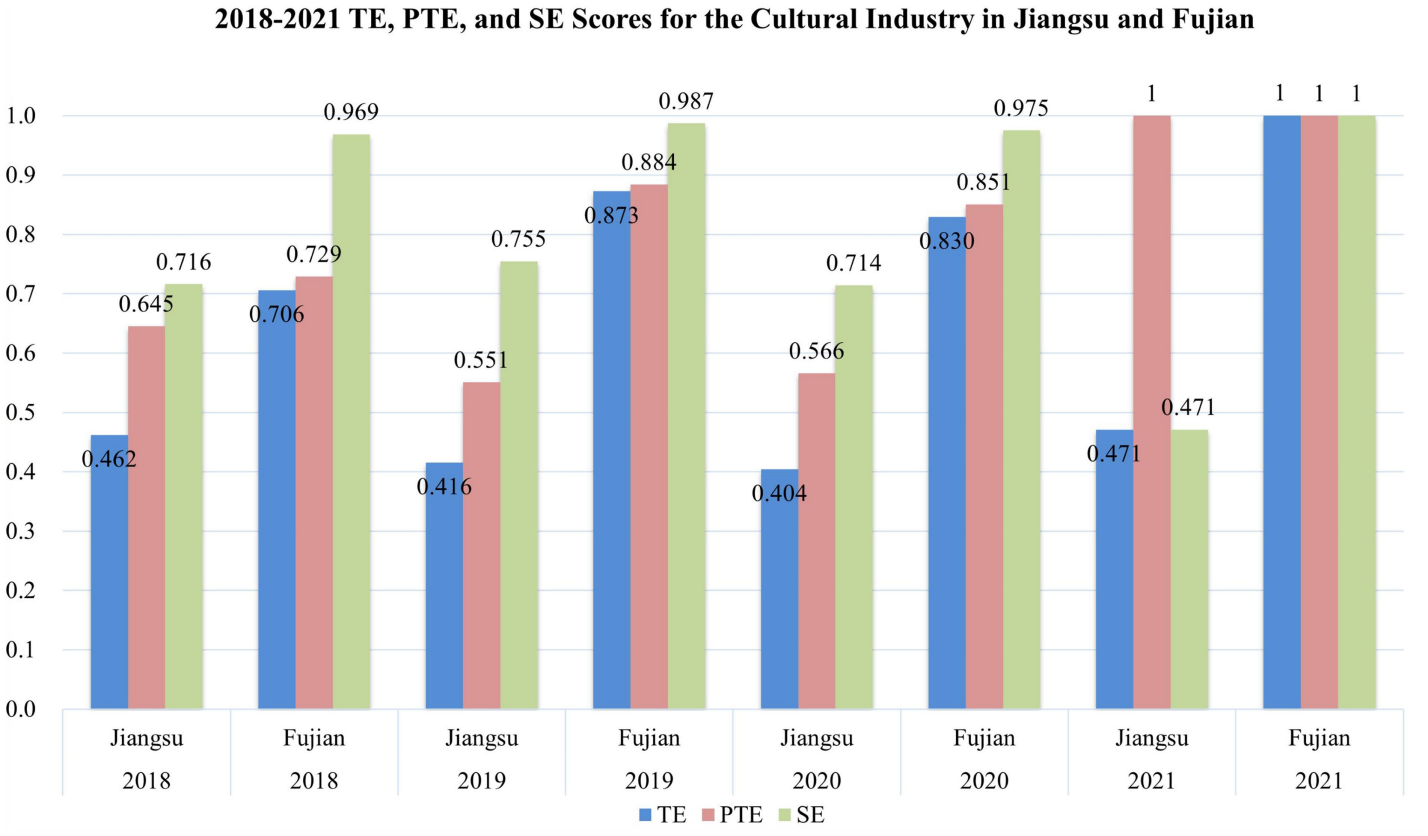
2018–2021 TE, PTE, and SE scores for the cultural industry in Jiangsu and Fujian.

Indeed, the total revenue of Jiangsu’s cultural industry is not low, as indicated in Figure 4, with rankings between 2nd and 3rd from 2018 to 2021. However, it must be emphasized again that the ultimate indicator for measuring the economic efficiency of the cultural industry is TE scores. Compared to Fujian, which ranks higher in TE, Jiangsu has not achieved more desirable outputs. High rankings in total revenue, combined with high rankings in asset scale, often conceal the reality of low efficiency and may lead decision-makers to overlook existing issues in Jiangsu’s cultural industry. The TE, PTE, and SE scores of Jiangsu and Fujian are summarized in Figure 6.

As evident from Figure 6, during the four periods studied, the scale efficiency of Jiangsu’s cultural industry is significantly lower than that of Fujian, particularly in 2021 where Jiangsu’s PTE score reached the DEA-effective level of 1, yet its scale efficiency drastically dropped to 0.471. In contrast, Fujian’s SE score consistently remained at or near the DEA-effective level of 1. When viewed in conjunction with Figure 2, Jiangsu’s scale efficiency is even much lower than the national average. The Return to Scale for Jiangsu and Fujian is summarized in Table 5 as follows.

**Table 5:** 2018–2021 return to scale for the cultural industry in Jiangsu and Fujian.

		Return to scale
2018	Jiangsu	DRS
	Fujian	DRS
2019	Jiangsu	DRS
	Fujian	DRS
2020	Jiangsu	DRS
	Fujian	DRS
2021	Jiangsu	DRS
	Fujian	CRS

When examining the Return of Scale, Jiangsu has been experiencing decreasing returns to scale for four consecutive years. This suggests that if the production scale is expanded further, the economic efficiency of Jiangsu’s cultural industry would decline even more. Decreasing returns to scale often arise due to diminishing marginal returns. The Austrian School’s Von Wieser established the theory of marginal utility (Von Wieser, 2013). As discussed earlier, Jiangsu’s input scale in the cultural industry has already reached the highest national level, typically indicating the onset of diminishing marginal returns. Taking tourism as an example, research by Jin et al. demonstrates that tourist attractions within Jiangsu are well-known (Jin and Tu, 2014). Data disclosed by the Suzhou city government indicates that in 2019 alone, the city of Suzhou in Jiangsu welcomed a staggering 136.09 million visitors (Suzhou, 2020). Overcapacity in visitor intake inevitably leads to reduced visitor satisfaction (Jin and Tu, 2014) and lower *per capita* spending (Liu et al., 2021). This manifests as diminishing marginal returns, causing a decline in Jiangsu’s scale efficiency and ultimately pulling down the overall efficiency of the cultural industry.

Despite Fujian’s efficiency scores being significantly higher than Jiangsu’s, it also experienced decreasing returns to scale for 3 years, only achieving constant returns to scale in 2021. This appears to be a common issue among economically developed provinces; other southeastern coastal provinces like Guangdong and Zhejiang have also been experiencing decreasing returns to scale for four consecutive years. This suggests that the challenges faced by Jiangsu’s cultural industry may be somewhat ubiquitous across China.

The global SBM model from 2018 to 2021 is input-oriented, thus its results also provide the slack of input variables for each province and city. The slack in input variables represents the surplus of inefficient inputs. To eliminate disparities among different provinces and cities, the slacks of input variables for Jiangsu and Fujian were normalized by dividing them by the respective input variable, and the results are summarized in Table 6. Throughout all periods, Jiangsu’s three input variables exhibit very high proportions of slack, whereas Fujian’s three variables have comparatively lower proportions of slack, all reaching zero by 2021.

**Table 6:** Comparison of input variable slack in the cultural industry of Jiangsu and Fujian from 2018 to 2021.

		Proportion of slack in the number of cultural industry enterprises	Proportion of slack in total assets of the cultural industry	Proportion of slack in the number of engaged persons in the cultural industry
2018	Jiangsu	69.65%	34.15%	57.55%
	Fujian	35.44%	0.00%	52.70%
2019	Jiangsu	70.16%	49.19%	55.94%
	Fujian	12.09%	0.00%	26.11%
2020	Jiangsu	67.40%	53.80%	57.50%
	Fujian	21.52%	0.00%	29.61%
2021	Jiangsu	59.08%	47.39%	52.19%
	Fujian	0.00%	0.00%	0.00%

As shown in Table 6, the highest proportion of input variable slack in Jiangsu is in the “Number of Enterprises,” with proportions exceeding 50% in all periods and reaching up to 70% at its peak. The next highest is “Engaged Persons at Year-end,” which also surpasses 50% in all periods. The slack proportion in “Total Assets” consistently exceeds 30%, with peaks over 50%. Such substantial slack is a direct cause of the low economic efficiency in Jiangsu’s cultural industry. The high proportion of slack in the number of enterprises and engaged persons, coupled with a relatively lower proportion of slack in the total assets of culturally based enterprises—which ranks first nationally—allows us to further infer: Compared to other provinces, the smaller scale of enterprises and excessive number of employed individuals are significant reasons for the inefficiency in Jiangsu’s cultural industry.

### 5.4 Expanding the scale of cultural enterprises in Jiangsu: a perspective from the Tobit model

From the perspective of the Tobit model, the inference drawn in Section 5.2 is further validated: Jiangsu’s cultural enterprises need to expand their scale. The variable ‘Per of assets of culture industry enterprises above scale’ has a significant positive influence on the economic efficiency of the cultural industry across provinces. Although Jiangsu ranks among the top in terms of the overall scale of the cultural industry nationwide, it ranks between 14th to 17th for the ‘Per of assets of culture industry enterprises above scale’ variable, as illustrated in Figure 6.

**Figure 7:**
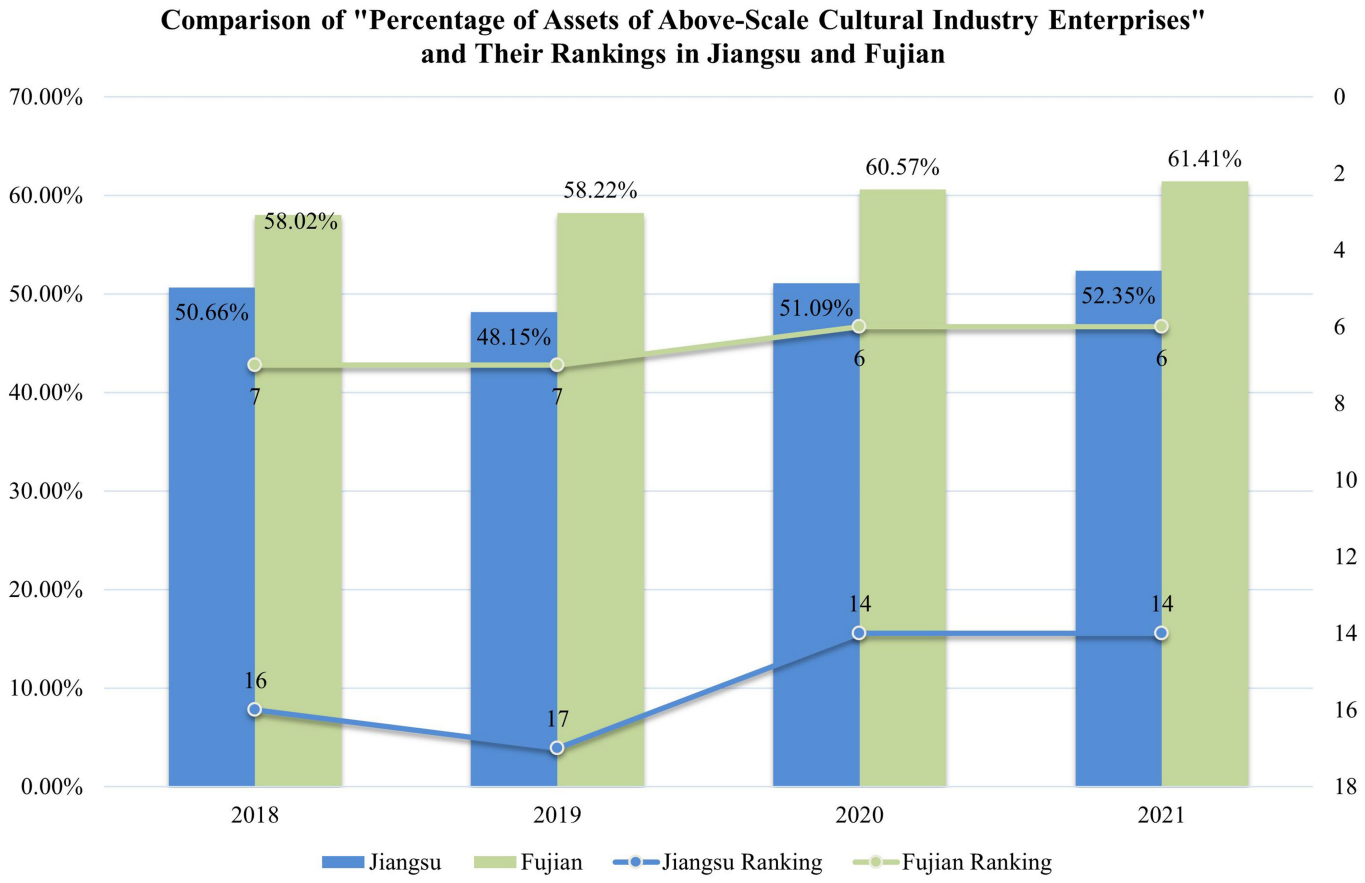
Comparison of “percentage of assets of above-scale cultural industry enterprises” and their rankings in Jiangsu and Fujian.

Jiangsu’s ranking in “Per of assets of culture industry enterprises above scale” indicates that the smaller scale of Jiangsu’s cultural enterprises is one of the significant factors impacting the economic efficiency of the province’s cultural industry. This conclusion aligns with the inferences drawn from the slack variable analysis in the SBM model described in Section 5.3. The governing authorities of the cultural industry in Jiangsu Province should place substantial emphasis on this aspect in the future (Figure 7).

In contrast, Fujian’s “Percentage of Assets of Above-Scale Cultural Industry Enterprises” and its ranking are significantly higher than those of Jiangsu, which is an important reason for the higher economic efficiency of Fujian’s cultural industry compared to Jiangsu.

### 5.5 The urbanization process in Jiangsu may exhibit certain inadequacies

**Figure 8:**
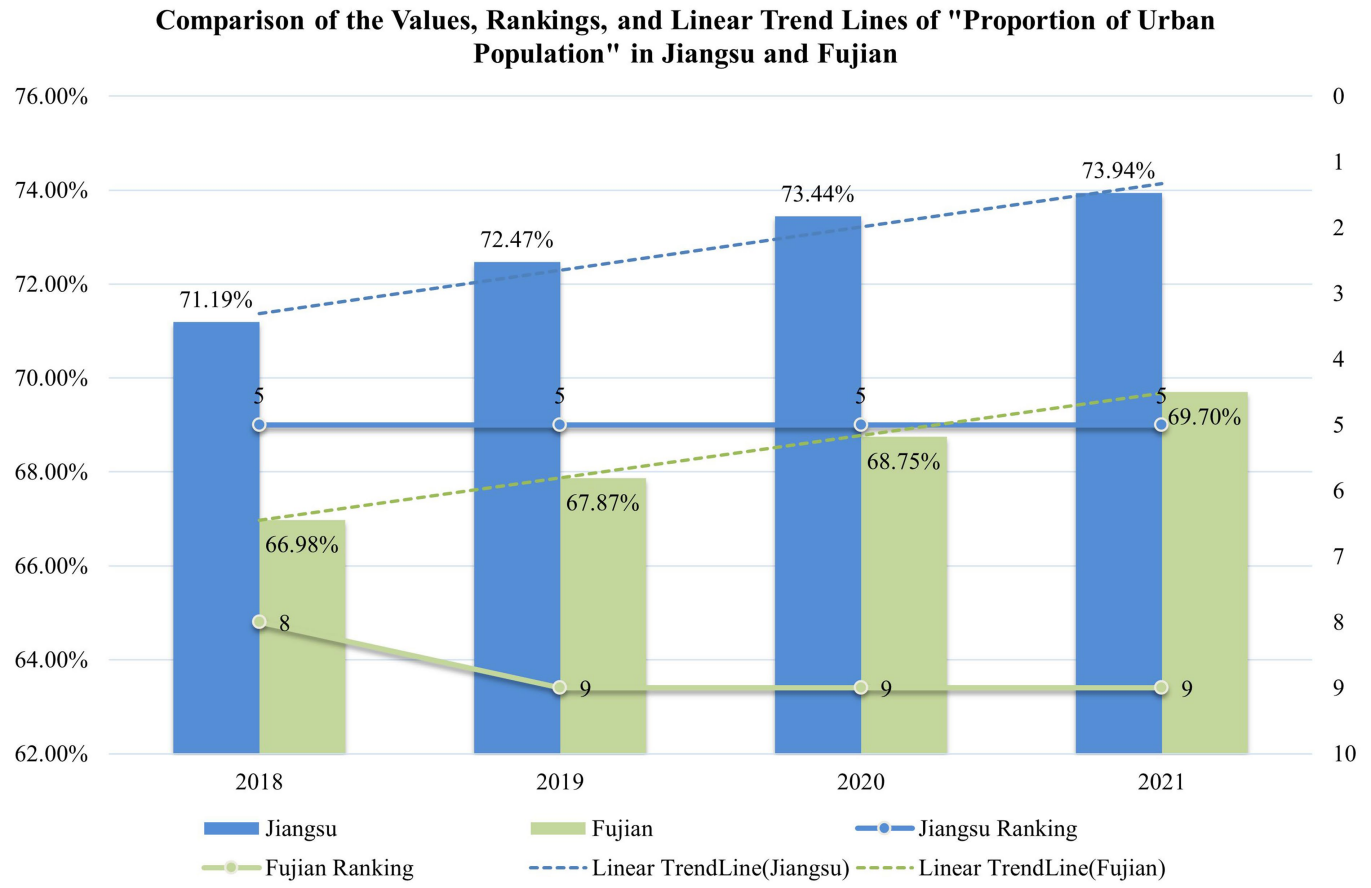
Proportion of Urban Population, rankings, and linear trends for Jiangsu and Fujian from 2018 to 2021.

The Tobit model also shows that the current economic efficiency of China’s cultural industry is significantly negatively affected by the “Proportion of Urban Population,” which represents the level of urbanization. As shown in Figure 8, both Jiangsu and Fujian are evaluated on their “Proportion of Urban Population.” This study includes the four municipalities of Beijing, Tianjin, Shanghai, and Chongqing, which typically have higher proportions of urban area and urban population compared to other provinces. In fact, Jiangsu, as a province, ranks 5th in “Proportion of Urban Population,” indicating that its level of urbanization is second only to Guangdong among all non-municipal provinces. Fujian, on the other hand, has significantly lower values and rankings than Jiangsu in this respect.

As shown in Figure 8, the trend lines for the “Proportion of Urban Population” in Jiangsu and Fujian have very similar slopes, with both increasing by nearly 3 percentage points during the study period. This suggests that the increases are the result of China’s unified and centralized new urbanization policies. In contrast, municipalities like Beijing and Shanghai, which already have high levels of urbanization, experienced far smaller changes in their “Proportion of Urban Population,” with an increase of less than one percentage point during the study period.

Sovani (1964) expressed concerns about “over-urbanization,” suggesting that many rural migrants are “pushed” rather than “pulled” into cities, leading to concurrent urban distress and rural poverty. Seto et al. (2010) argue that, within the context of sustainable development, the characteristics of contemporary urbanization, along with urban planning, governance, agglomeration, and the forces of globalization, play roles in driving and shaping the relationship between urbanization and the environment, which significantly differ from traditional urbanization. Chan (2010) conducted a study on China’s urbanization policies, describing previous urbanization efforts as “incomplete urbanization.”

Research by Chaolin et al. (2012) indicates that studies on urbanization in China face issues such as a vague understanding of the urbanization concept, a lack of fundamental statistics, a focus on empirical phenomena over theoretical exploration, and an emphasis on regional characteristics. Guan et al. (2018) suggest that the traditional process of urbanization in China is increasingly unsustainable, and there is an urgent need to accelerate the transformation toward a people-oriented new model of urbanization, which is intensive, efficient, integrates urban and rural areas, and is harmonious and sustainable. Ni (2023) observed in their study of Suzhou and Yancheng in Jiangsu that one of the main methods of urbanization has been the reclassification of counties to districts, converting large areas previously considered rural into urban. This form of urbanization, perceived as a legitimate means of fostering local growth and forming effective, state-centered governance, aligns with the characteristics of China’s urbanization, social progress, political system, and development model. However, this could lead to decentralized governance, potentially impacting economic efficiency, and necessitates ongoing, reflective research.

Consequently, the suboptimal economic efficiency of Jiangsu’s cultural industry is influenced by the province’s unreasonable urbanization processes, whereas Fujian, with a lower degree of urbanization, faces relatively minor negative impacts. Recent studies (Zhu et al., 2023) suggest that the construction of culturally-oriented cities can help reconcile the contradictions between excessive urbanization and economic efficiency. Actively developing and optimizing the cultural industry may ultimately accommodate the outcomes of rapid urbanization, creating a win-win situation.

In summary, Hypothesis 3 of this study is rejected, suggesting that the current comprehensive policies in Jiangsu Province do not fully favor the healthy development of the cultural industry and require appropriate adjustments.

## 6 Conclusion and recommendations

### 6.1 Conclusion

- Based on the discussions presented earlier, the economic efficiency of China’s cultural industry is on a generally positive trajectory. Despite being affected by COVID-19, a significant recovery can be observed.
- Despite the substantial investment and attention given to the cultural industry in Jiangsu, its economic efficiency remains suboptimal at the national level.
- As deduced from Section 5.3, when compared to other provinces, a salient issue with Jiangsu’s cultural industry is its lack of scale efficiency. Further analysis indicates that Jiangsu’s cultural industry is experiencing diminishing scale returns, meaning that increasing investments may ironically lead to a decline in economic efficiency.
- Drawing from the slack variable analysis in Section 5.3 and the influencing factor analysis in Section 5.5, a primary reason for Jiangsu’s cultural industry’s low economic efficiency is the smaller scale of cultural enterprises in the province and their inherent low operational efficiency.
- Integrating the above conclusions with the insights from Section 5.6 and related literature, this study contends that the economic efficiency of Jiangsu’s cultural industry is constrained by its urbanization development. While Jiangsu’s urbanization rate is among the leaders nationally, its reliance on administrative measures like de-county and re-districting to propel its urbanization suggests that the province is still grappling with the aftermath of such rapid development, leading to relatively lower economic efficiency in its cultural industry.

### 6.2 Recommendations

Based on the above conclusions, this study recommends the following policy considerations for the future development of Jiangsu’s cultural industry:

- There should be greater encouragement for the structural adjustment of the cultural industry, while being particularly cautious about expanding the scale of industry investments.
- The development of leading cultural enterprises in Jiangsu should be actively supported and encouraged, with a keen emphasis on facilitating mergers and optimizations among similar enterprises.
- China’s official definition of the cultural industry is broad, encompassing sectors such as news, publishing, film and television, creativity, entertainment services, tourism, related manufacturing, related wholesale and retail, and so forth. For Jiangsu to enhance its economic efficiency in the cultural industry, it should proactively seek new industry directions that offer higher efficiency and returns.
- Learning from provinces and cities with relatively higher economic efficiency in the cultural industry, like Shanghai, can provide valuable insights and advanced practices to be adopted.

## Data Availability

The original contributions presented in the study are included in the article/supplementary material, further inquiries can be directed to the corresponding author/s.
