# Supplementary material for: An integrative study on the green cultural industry and its determinants in Jiangsu province, China under the cultural revitalization initiative: a global perspective
Source: Front Psychol. 2024 Nov 12;15:1328121. doi: 10.3389/fpsyg.2024.1328121 (PMC11601999; doi:10.3389/fpsyg.2024.1328121)
Supplement: Supplementary file 1 [file Table_1.DOCX]

Supplementary Material

Table S1. List of 2018-2021 China's 31 Provinces and Cities Culture Industry Economic Efficiency Global SBM Model

| **Period** | **DMU** | **TE** | **PTE** | **SE** | **Return of scale** |
| --- | --- | --- | --- | --- | --- |
| 2018 | Beijing | 0.560576 | 0.662762 | 0.845818 | DRS |
| 2018 | Tianjin | 0.575284 | 0.576238 | 0.998345 | IRS |
| 2018 | Hebei | 0.219723 | 0.233517 | 0.94093 | DRS |
| 2018 | Shanxi | 0.140621 | 0.140621 | 1 | CRS |
| 2018 | Inner Mongolia | 0.110093 | 0.110093 | 1 | CRS |
| 2018 | Liaoning | 0.257673 | 0.267067 | 0.964823 | DRS |
| 2018 | Jilin | 0.153718 | 0.153718 | 1 | CRS |
| 2018 | Heilongjiang | 0.190691 | 0.190691 | 1 | CRS |
| 2018 | Shanghai | 1 | 1 | 1 | CRS |
| 2018 | Jiangsu | 0.46215 | 0.645335 | 0.716139 | DRS |
| 2018 | Zhejiang | 0.556347 | 0.621231 | 0.895556 | DRS |
| 2018 | Anhui | 0.44194 | 0.46497 | 0.950471 | DRS |
| 2018 | Fujian | 0.706209 | 0.729001 | 0.968736 | DRS |
| 2018 | Jiangxi | 0.459893 | 0.472015 | 0.974319 | DRS |
| 2018 | Shandong | 0.439348 | 0.453079 | 0.969694 | DRS |
| 2018 | Henan | 0.404806 | 0.432008 | 0.937033 | DRS |
| 2018 | Hubei | 0.408278 | 0.417137 | 0.978763 | DRS |
| 2018 | Hunan | 0.51058 | 0.523868 | 0.974633 | DRS |
| 2018 | Guangdong | 0.568301 | 1 | 0.568301 | DRS |
| 2018 | Guangxi | 0.220997 | 0.226689 | 0.974889 | DRS |
| 2018 | Hainan | 0.222798 | 0.225616 | 0.98751 | IRS |
| 2018 | Chongqing | 0.397619 | 0.408703 | 0.97288 | DRS |
| 2018 | Sichuan | 0.402424 | 0.409521 | 0.982669 | DRS |
| 2018 | Guizhou | 0.16092 | 0.161166 | 0.998473 | DRS |
| 2018 | Yunnan | 0.237151 | 0.24389 | 0.972366 | DRS |
| 2018 | Tibet | 0.432469 | 1 | 0.432469 | IRS |
| 2018 | Shaanxi | 0.237216 | 0.242376 | 0.978713 | DRS |
| 2018 | Gansu | 0.116028 | 0.116028 | 1 | CRS |
| 2018 | Qinghai | 0.179792 | 0.787774 | 0.228227 | IRS |
| 2018 | Ningxia | 0.159757 | 0.555449 | 0.287617 | IRS |
| 2018 | Xinjiang | 0.325403 | 0.328097 | 0.991791 | DRS |
| 2019 | Beijing | 0.603791 | 0.830584 | 0.726948 | DRS |
| 2019 | Tianjin | 0.654445 | 0.667565 | 0.980346 | DRS |
| 2019 | Hebei | 0.227499 | 0.240112 | 0.947473 | DRS |
| 2019 | Shanxi | 0.127021 | 0.127021 | 1 | CRS |
| 2019 | Inner Mongolia | 0.135093 | 0.135093 | 1 | CRS |
| 2019 | Liaoning | 0.246621 | 0.255654 | 0.964668 | DRS |
| 2019 | Jilin | 0.161065 | 0.161715 | 0.995978 | IRS |
| 2019 | Heilongjiang | 0.206829 | 0.206829 | 1 | CRS |
| 2019 | Shanghai | 0.848711 | 0.848711 | 1 | CRS |
| 2019 | Jiangsu | 0.415681 | 0.5507 | 0.754823 | DRS |
| 2019 | Zhejiang | 0.583158 | 0.710027 | 0.821318 | DRS |
| 2019 | Anhui | 0.388836 | 0.402104 | 0.967004 | DRS |
| 2019 | Fujian | 0.872677 | 0.884107 | 0.987072 | DRS |
| 2019 | Jiangxi | 0.480536 | 0.486026 | 0.988703 | DRS |
| 2019 | Shandong | 0.467464 | 0.467464 | 1 | CRS |
| 2019 | Henan | 0.451315 | 0.485206 | 0.930152 | DRS |
| 2019 | Hubei | 0.417561 | 0.423704 | 0.9855 | DRS |
| 2019 | Hunan | 0.513604 | 0.526241 | 0.975986 | DRS |
| 2019 | Guangdong | 0.535648 | 0.900842 | 0.594608 | DRS |
| 2019 | Guangxi | 0.350037 | 0.365715 | 0.95713 | DRS |
| 2019 | Hainan | 0.214882 | 0.216812 | 0.991096 | IRS |
| 2019 | Chongqing | 0.382153 | 0.39446 | 0.9688 | DRS |
| 2019 | Sichuan | 0.446784 | 0.456671 | 0.97835 | DRS |
| 2019 | Guizhou | 0.166894 | 0.167143 | 0.998511 | IRS |
| 2019 | Yunnan | 0.296071 | 0.307412 | 0.963108 | DRS |
| 2019 | Tibet | 1 | 1 | 1 | CRS |
| 2019 | Shaanxi | 0.234942 | 0.241027 | 0.974754 | DRS |
| 2019 | Gansu | 0.135177 | 0.135177 | 1 | CRS |
| 2019 | Qinghai | 0.160225 | 1 | 0.160225 | IRS |
| 2019 | Ningxia | 0.181111 | 1 | 0.181111 | IRS |
| 2019 | Xinjiang | 0.305923 | 0.309098 | 0.989728 | DRS |
| 2020 | Beijing | 0.612394 | 0.898468 | 0.681598 | DRS |
| 2020 | Tianjin | 0.572128 | 0.593153 | 0.964554 | DRS |
| 2020 | Hebei | 0.251541 | 0.26495 | 0.94939 | DRS |
| 2020 | Shanxi | 0.131607 | 0.131607 | 1 | CRS |
| 2020 | Inner Mongolia | 0.137942 | 0.137942 | 1 | CRS |
| 2020 | Liaoning | 0.229064 | 0.237364 | 0.965029 | DRS |
| 2020 | Jilin | 0.17048 | 0.180702 | 0.943434 | IRS |
| 2020 | Heilongjiang | 0.178911 | 0.178911 | 1 | CRS |
| 2020 | Shanghai | 0.783817 | 0.82232 | 0.953178 | DRS |
| 2020 | Jiangsu | 0.40435 | 0.566045 | 0.714342 | DRS |
| 2020 | Zhejiang | 0.601128 | 0.800093 | 0.751322 | DRS |
| 2020 | Anhui | 0.394757 | 0.407323 | 0.969151 | DRS |
| 2020 | Fujian | 0.82956 | 0.850691 | 0.97516 | DRS |
| 2020 | Jiangxi | 0.490601 | 0.490619 | 0.999963 | CRS |
| 2020 | Shandong | 0.530295 | 0.530295 | 1 | CRS |
| 2020 | Henan | 0.431152 | 0.459492 | 0.938323 | DRS |
| 2020 | Hubei | 0.390426 | 0.397016 | 0.983402 | DRS |
| 2020 | Hunan | 0.483319 | 0.493961 | 0.978456 | DRS |
| 2020 | Guangdong | 0.490702 | 0.823679 | 0.595744 | DRS |
| 2020 | Guangxi | 0.366687 | 0.378919 | 0.967719 | DRS |
| 2020 | Hainan | 0.34087 | 0.342416 | 0.995485 | IRS |
| 2020 | Chongqing | 0.371761 | 0.380704 | 0.97651 | DRS |
| 2020 | Sichuan | 0.433827 | 0.439269 | 0.987612 | DRS |
| 2020 | Guizhou | 0.169936 | 0.171116 | 0.993106 | IRS |
| 2020 | Yunnan | 0.28005 | 0.288249 | 0.971555 | DRS |
| 2020 | Tibet | 0.897576 | 0.897576 | 1 | CRS |
| 2020 | Shaanxi | 0.214519 | 0.220586 | 0.972498 | DRS |
| 2020 | Gansu | 0.138337 | 0.138337 | 1 | CRS |
| 2020 | Qinghai | 0.076477 | 0.185988 | 0.411194 | IRS |
| 2020 | Ningxia | 0.17698 | 1 | 0.17698 | IRS |
| 2020 | Xinjiang | 0.273315 | 0.27647 | 0.988587 | DRS |
| 2021 | Beijing | 0.629909 | 1 | 0.629909 | DRS |
| 2021 | Tianjin | 0.499483 | 0.523796 | 0.953581 | DRS |
| 2021 | Hebei | 0.299647 | 0.313606 | 0.95549 | DRS |
| 2021 | Shanxi | 0.183716 | 0.1845 | 0.995747 | DRS |
| 2021 | Inner Mongolia | 0.166216 | 0.166216 | 1 | CRS |
| 2021 | Liaoning | 0.260706 | 0.271762 | 0.959319 | DRS |
| 2021 | Jilin | 0.249617 | 1 | 0.249617 | IRS |
| 2021 | Heilongjiang | 0.196469 | 0.196469 | 1 | CRS |
| 2021 | Shanghai | 1 | 1 | 1 | CRS |
| 2021 | Jiangsu | 0.471123 | 1 | 0.471123 | DRS |
| 2021 | Zhejiang | 0.658982 | 1 | 0.658982 | DRS |
| 2021 | Anhui | 0.421367 | 0.43144 | 0.976652 | DRS |
| 2021 | Fujian | 1 | 1 | 1 | CRS |
| 2021 | Jiangxi | 0.56931 | 0.569356 | 0.99992 | CRS |
| 2021 | Shandong | 1 | 1 | 1 | CRS |
| 2021 | Henan | 0.494026 | 0.526501 | 0.938319 | DRS |
| 2021 | Hubei | 0.486251 | 0.49453 | 0.983259 | DRS |
| 2021 | Hunan | 0.529329 | 0.539623 | 0.980924 | DRS |
| 2021 | Guangdong | 0.557939 | 1 | 0.557939 | DRS |
| 2021 | Guangxi | 0.384317 | 0.389465 | 0.986781 | DRS |
| 2021 | Hainan | 0.460764 | 0.461084 | 0.999305 | IRS |
| 2021 | Chongqing | 0.447663 | 0.456722 | 0.980165 | DRS |
| 2021 | Sichuan | 0.451113 | 0.452611 | 0.99669 | DRS |
| 2021 | Guizhou | 0.191303 | 0.193502 | 0.988636 | IRS |
| 2021 | Yunnan | 0.276466 | 0.281608 | 0.981743 | DRS |
| 2021 | Tibet | 0.856159 | 0.856159 | 1 | CRS |
| 2021 | Shaanxi | 0.194457 | 0.198707 | 0.978612 | DRS |
| 2021 | Gansu | 0.162227 | 0.162227 | 1 | CRS |
| 2021 | Qinghai | 0.078459 | 0.190425 | 0.412021 | IRS |
| 2021 | Ningxia | 0.230534 | 1 | 0.230534 | IRS |
| 2021 | Xinjiang | 0.235855 | 0.238558 | 0.988673 | DRS |
